# EasyCluster: a fast and efficient gene-oriented clustering tool for large-scale transcriptome data

**DOI:** 10.1186/1471-2105-10-S6-S10

**Published:** 2009-06-16

**Authors:** Ernesto Picardi, Flavio Mignone, Graziano Pesole

**Affiliations:** 1 Dipartimento di Biochimica e Biologia Molecolare "E. Quagliariello", Università degli Studi di Bari, 70126 Bari, Italy; 2 Dipartimento di Chimica Strutturale e Stereochimica Inorganica, Università degli Studi di Milano, 20133 Milano, Italy; 3 Istituto Tecnologie Biomediche del Consiglio Nazionale delle Ricerche, via Amendola 122/D, 70125 Bari, Italy

## Abstract

**Background:**

ESTs and full-length cDNAs represent an invaluable source of evidence for inferring reliable gene structures and discovering potential alternative splicing events. In newly sequenced genomes, these tasks may not be practicable owing to the lack of appropriate training sets. However, when expression data are available, they can be used to build EST clusters related to specific genomic transcribed *loci*. Common strategies recently employed to this end are based on sequence similarity between transcripts and can lead, in specific conditions, to inconsistent and erroneous clustering. In order to improve the cluster building and facilitate all downstream annotation analyses, we developed a simple genome-based methodology to generate gene-oriented clusters of ESTs when a genomic sequence and a pool of related expressed sequences are provided. Our procedure has been implemented in the software EasyCluster and takes into account the spliced nature of ESTs after an *ad hoc *genomic mapping.

**Methods:**

EasyCluster uses the well-known GMAP program in order to perform a very quick EST-to-genome mapping in addition to the detection of reliable splice sites. Given a genomic sequence and a pool of ESTs/FL-cDNAs, EasyCluster starts building genomic and EST local databases and runs GMAP. Subsequently, it parses results creating an initial collection of pseudo-clusters by grouping ESTs according to the overlap of their genomic coordinates on the same strand. In the final step, EasyCluster refines the clustering by again running GMAP on each pseudo-cluster and groups together ESTs sharing at least one splice site.

**Results:**

The higher accuracy of EasyCluster with respect to other clustering tools has been verified by means of a manually cured benchmark of human EST clusters. Additional datasets including the Unigene cluster Hs.122986 and ESTs related to the human *HOXA *gene family have also been used to demonstrate the better clustering capability of EasyCluster over current genome-based web service tools such as ASmodeler and BIPASS. EasyCluster has also been used to provide a first compilation of gene-oriented clusters in the *Ricinus communis *oilseed plant for which no Unigene clusters are yet available, as well as an evaluation of the alternative splicing in this plant species.

## Background

Expressed sequence tags are short single pass randomly selected sequence reads derived from cDNA libraries. Despite their error prone nature, ESTs harbour several advantages and to date have been used for a plethora of purposes including gene discovery and annotation, identification of genetic variations as single nucleotide polymorphisms (SNPs) and detection of splice variants (for a comprehensive review see [1] and [2]). Moreover, ESTs have been also fruitfully exploited in genome mapping and gene expression studies.

However, next generation sequencing strategies, providing a drastic increase in sequencing capacity, are quickly emerging as worthy alternatives to EST projects and promise great improvements in understanding of the complexity of eukaryotic transcriptomes [3]. Short reads, in fact, allow more accurate gene expression profiles and simplify the identification of transcribed *loci *appearing as waves of reads along a reference genome. Nevertheless, interest in ESTs has not decreased as evidenced by the exponential growth of such sequences in specialized databases such as dbEST at NCBI [4]. In contrast to short reads, ESTs can be efficiently mapped on reference genomes providing indispensable and invaluable sources of evidence for inferring reliable gene structures and discovering potential alternative splicing events. Their contribution is particularly relevant and important in newly sequenced genomes in which these tasks could not be feasible for several limitations such as the lack of appropriate training sets. For this reason, ESTs have been successfully embedded as a principal evidence source in different *ab initio *gene prediction tools as AUGUSTUS [5], TWINSCAN_EST [6] and N_SCAN_EST [7] or employed as such to assemble complete gene structures by directed acyclic graphs and deduce alternative splicing events through *ad hoc *computational tools such as Exogean [8] and ASPIC [9].

The recent publication of different web services as EST2uni [10], ESTpass [11], ESTExplorer [12] or EGassembler [13] providing more or less complex pipelines to handle and analyse ever greater amount of ESTs and FL-cDNAs also attest to the evergreen interest in these single pass cDNA reads. These pipelines include facilities and well-established tools to clean ESTs, removing genomic and vector contaminants as well as polyA tails, and produce tentative functional annotations by means of the combination of programs as BLAST [14], InterPro [15] and CAP3 [16].

Without any doubt one of the most important issues concerning the analysis of expressed sequence tags and FL-cDNAs is the generation of gene-oriented clusters or, in other terms, groups of overlapping ESTs from the same gene *locus*. Several tools have been designed to this end as transcript clustering is an essential step for many bioinformatics analyses. However, the clustering of large collections of ESTs has very high requirements both in terms of memory usage and computational power.

The majority of EST clustering algorithms developed until now rely on pairwise comparisons between ESTs. Such software, however, works efficiently with small samples of sequences because of the *O*(*n*^2^) computational complexity. To solve this task different strategies have been proposed. The program CLOBB, for example, builds EST clusters using BLAST searches over local EST databases [17]. Recently, a modified version of BLAST named BLASTClust, available at NCBI web site, has been introduced to improve the clustering of biological sequences according to the existence of significant local similarities [18]. BLASTClust formats input sequences to produce temporary BLAST databases and performs clustering using the BLASTp algorithm in case of protein sequences or the megaBLAST algorithm in case of nucleotide sequences. The quality of the resulting clusters is however strongly biased by the choice of optimal BLAST parameters to control the stringency of clustering including thresholds for score density, percent identity, and alignment length.

Another well-established program to group ESTs sharing significant regions of near identity is TGICL [19], developed at the TIGR Institute and currently used to build "strict" clusters of similar ESTs that are then used in the construction of tentative consensus sequences to be collected in the TIGR Gene Index database [20]. TGICL is based on mgBLAST, a modified version of megaBLAST that provides additional output filtering and quickly performs all-to-all pairwise comparisons between EST sequences.

A complementary approach is taken to populate the well-known UniGene database at NCBI [21] where high quality ESTs are grouped in clusters taking into account the sequence overlap above a given alignment threshold according to a "transcript based" approach based on megaBLAST. When reliable genome assemblies are available, UniGene clusters are generated following a "genome based" strategy in which exon/intron boundaries of ESTs aligned onto a reference genome are taken into account during the cluster building.

The method used to build the STACK database [22] is quite different from the previously described approaches, even though it still requires pairwise comparisons between ESTs. Instead of using a local BLAST-like alignment algorithm, it considers two ESTs to be closely related if a sufficiently large percentage of bases are identical within a fixed-length sliding window. Resulting clusters, sometimes defined "loose", are then assembled by Phrap program [23] before the inclusion in the database. Very recently a new version of this algorithm has been implemented in the wcd [24] program – optionally replacing the d2_cluster [25] software within the StackPack system [2].

Suffix tree based approaches have also been used to speed up the identification of significant overlapping regions of pairwise ESTs with satisfactory results. Successful implementations of suffix tree algorithms can be found in the programs PaCE [26] and ClustDB [27].

Although different implementations of similarity-based methods exist, they share several limitations that frequently lead to inconsistent clusters. ESTs from paralogous genes or overlapping genes on opposite strands, for example, may not be adequately separated and placed in different clusters. For these reasons and in order to create more gene-oriented EST clusters, genomic information has been taken into account when possible. In BIPASS [28], a recent web service resource, input ESTs are first mapped onto the corresponding genome by Blat [29] and resulting alignments are refined by sim4 program [30]. Next, overlapping ESTs are grouped in the same cluster. A similar approach is taken by ASmodeler [31] in which Blat and sim4 are again used to map and align ESTs onto a reference genome and drive a first clustering of overlapping ESTs according to detected coordinates. Then, each cluster is refined taking into account exon/intron boundaries.

EST clusters generated employing genome information are especially useful to improve the identification of expressed gene *loci *and infer alternative splicing events. However, dedicated software to efficiently generate genome-based clusters of ESTs is not yet available. BIPASS and ASmodeler, for example, are web services able to analyze only limited amount of ESTs and in general cannot be used with newly sequenced genomes. On the other hand, the software used to create genome-based UniGene clusters is not currently publicly available at NCBI.

In order to fill this gap, improve existing procedures for cluster building and facilitate downstream and annotation analyses in new genomes, we developed an easy but efficient system to generate UniGene-like clusters of ESTs when a genomic sequence and a pool of related expressed sequences are provided. Our procedure, implemented in the program hereafter named EasyCluster, takes into account the spliced nature of ESTs after an *ad hoc *genomic mapping. In particular, EasyCluster first performs an EST to genome mapping using the well-established GMAP program [32] and then groups aligned ESTs according to the biological assumption that two or more ESTs belong to the same transcribed *locus *if they share at least one splice site. EasyCluster also implements the detection of alternative splicing events and provides graphical overviews of detected clusters in pure HTML format. Moreover, it has been devised to handle large numbers of ESTs and complete genomes on desktop computers.

The reliability of EasyCluster has been assessed using a manually curated human benchmark of more 17,000 ESTs and results have been compared with those obtained using other recent programs for EST clustering. Finally, the application of EasyCluster to the oilseed plant *Ricinus communis*, for which a draft genome sequence but no UniGene clusters are available, provided a first set of 5,879 gene-oriented clusters predicting at least 918 alternative splicing events.

## Methods

### Algorithm description

**Figure 1:**
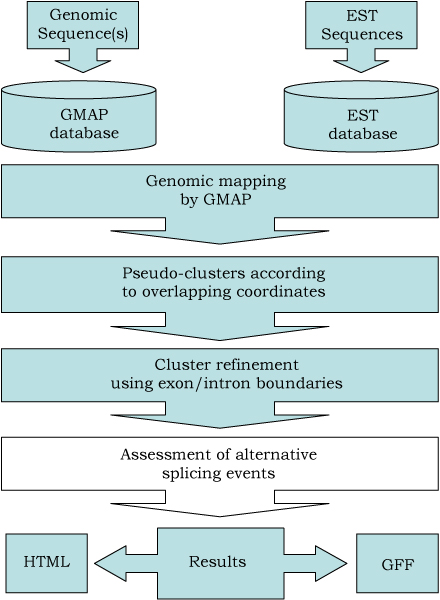
**Graphical overview of EasyCluster algorithm and work-flow**. In EasyCluster, genomic and EST sequences are initially used to build local databases. Next, GMAP is used to produce EST to genome alignments and results are parsed to build a first round of pseudo-clusters according to overlapping coordinates. For each cluster a refinement procedure is used to generate final clusters taking into account exon/intron boundaries. Before results, the prediction of alternative splicing events per cluster can be optionally required.

The algorithm implemented in EasyCluster harbours new and unique features in order to improve the clustering process and, at the same time, facilitate the generation of gene-oriented clusters to researchers without advanced skills in bioinformatics. EasyCluster, in fact, can be used interactively providing only two Fasta files containing genomic and EST sequences, respectively. The main steps of the algorithm are shown in the flow chart in Figure 1 and can be summarized in the following six points:

#### 1. Building GMAP and EST databases

The goal of this first step is to read input Fasta files and store sequences in *ad hoc *local databases increasing memory efficiency and making available the management of complete genomes on desktop computers. In case of genomic sequences, EasyCluster uses the GMAP_SETUP facility to construct a genomic oligomer index and a genomic sequence file in binary format starting from an arbitrary number of chromosomes or contigs. This pre-processing phase is particularly useful to speed up GMAP mapping, reduce memory requirements (minimum is only 128 MB) even for large complete genomes and provide rapid access to any genomic sub-sequence.

In case of ESTs and FL-cDNAs, EasyCluster employs the *anydbm *python module to builds a local database of expressed sequences. Such database is like a dictionary in which each key is the name of the EST (the header of each Fasta sequence) and the corresponding value is the nucleotide sequence. When the number of ESTs is large, the program makes use of the C library *DNA_Stat *[33] through the *ctypes *python module (embedded in python version 2.5 and higher). The *DNA_Stat *library is currently part of the ClustDB software by Kleffe et al. [27], one the fastest programs to identify groups of similar nucleotide sequences by suffix trees. In EasyCluster, the *DNA_Stat *library is used instead of the *anydbm *module to save disk space and improve sequence retrieval.

Local GMAP and EST databases can also be used to extract specific genomic sub-regions or retrieve lists of EST sequences according to their accessions by means of an accessory python script called *getFastas.py *that is part of the current EasyCluster release. If such databases have been previously created, they can directly used in EasyCluster providing the corresponding name and hard drive location. This option is particularly useful for complete genomes and avoids performing the database building step every time EasyCluster is launched.

#### 2. GMAP mapping

In this step EasyCluster runs GMAP in order to quickly map ESTs onto a given genomic sequence – identifying canonical and non-canonical splice sites in parallel. Moreover, GMAP is not oriented to a specific organism and can be successfully used in both animal and plant genomes. GMAP allows the detection of microexons, undetectable by similar software, and automatically trims low quality sequences containing polyA tails or vector contaminants. EST sequences resulting in poor genome alignments are excluded from mapping. As a consequence, EasyCluster can handle ESTs of different quality without specific pre-processing steps. However, clusters produced using low quality ESTs should be carefully inspected before any downstream use. Generally, the main effect of low quality ESTs is an increased number of singletons (clusters containing only one EST).

A desirable feature of GMAP is also the production of a compressed output file with specific indications about mapping coordinates, EST orientation, percentage of alignment identity and coverage. In addition, GMAP can optionally speed up the EST to genome mapping depending on available RAM memory or CPUs thanks to its multithreading architecture.

#### 3. Generation of pseudo-clusters

After the EST to genome mapping, GMAP results are parsed in order to keep only significant ESTs according to two simple filtering criteria based on the minimum percent identity and the minimum percent of alignment coverage. Moreover, only sequences with multiple exons (spliced) are retained for downstream analyses. This procedure notably reduces unwanted sequences such as genomic DNA contaminants that generally represent a major obstacle in EST data processing. Sometimes, single ESTs can map on different genomic locations. In these cases, EasyCluster takes only sequences showing the highest values of alignment identity and coverage. In rare cases of more than one identical mapping score, all alternative paths are included in the clustering process.

Filtered ESTs are split taking into account the reference genomic region and the strand. All ESTs are finally sorted according to mapping coordinates and overlapping sequences are grouped in pseudo-clusters.

#### 4. Cluster refinement

The EST clustering process is complicated by the lack of information about the gene structures for some gene *loci *– even in well-annotated genomes where many protein products are known. This prevents the generation of accurate gene-oriented clusters even when the gene is fully covered by at least one cDNA/EST sequence. Moreover, the clustering efficiency decreases when only EST sequences are available. However, a way to reduce wrong clustering is to introduce a stringent but biologically reasonable criterion based on exon/intron borders. For this reason, EasyCluster runs GMAP again on each pseudo-cluster and resulting aligned ESTs sharing at least one splice site are grouped together. The use of GMAP on pseudo-clusters and thus on genomic sub-regions increases the quality of EST to genome alignments improving the detection of real splice sites.

Moreover, the biological constraint introduced makes EasyCluster more reliable than BIPASS where only overlapping coordinates are used to cluster related ESTs. ASmodeler, instead, is based on the same biological criterion even though the clustering is less stringent – it assumes that neighbouring splice sites are considered identical if they are within ± 16 base pairs.

EST clusters generated in this phase are ready for transcript assembly, alternative splicing prediction, gene discovery and annotation.

#### 5. Prediction of alternative splicing events

Once EST gene-oriented clusters have been generated, EasyCluster can optionally deduce alternative splicing events thanks to an appropriate module implementing a modified version of the ASTALAVISTA algorithm [34]. According to this methodology, all pairwise comparisons between overlapping ESTs of each cluster are initially considered. Subsequently, taking into account the genomic coordinates of mapped ESTs, variations of the splicing structure are detected every time some splice sites are not used in both sequences. Such variations are finally exploited to build a code describing the corresponding alternative splicing events. EasyCluster reads this code and returns basic statistics about the impact of alternative splicing both globally and for each genomic region or cluster.

#### 6. Report and clustering results

The presentation of results is a crucial consideration for all bioinformatics tools and user friendly and widespread formats are generally desirable. In order to make EasyCluster results useful for subsequent computational analyses, the program generates two text files, one in tabular format with a cluster per line and the other in the general feature format (see GFF standards at [35]) with mapping details for each EST of a cluster. Moreover, EasyCluster provides a main report page in HTML format to simplify the interpretation of results and to explore different steps of the clustering procedure. Specific tables are generated to view input parameters, examine the time taken to complete each algorithm step, obtain main GMAP statistics and graphical distributions of EST percent identity and coverage. EasyCluster also provides graphical overviews for each generated cluster, giving the opportunity to rigorously browse individual EST groups in their genomic contest and off-line. All result web pages are readable by standard browsers. However, FireFox 1.5.x or superior is recommended since cluster graphs are drawn using CSS code optimized for FireFox that might not be stable with all web browsers.

### Datasets

Three main datasets have been used to test EasyCluster: B1) the UniGene cluster Hs.122986 corresponding to the *TPTE *gene; B2) the full set of transcripts/ESTs mapping on the region of human chromosome 7 that contains the *HOXA *gene family; B3) a manually curated benchmark of human gene-oriented transcript clusters. The UniGene cluster was directly retrieved from the NCBI web site through the Entrez system. For the human *HOXA *gene family, human spliced ESTs, FL-cDNAs and RefSeqs were downloaded from UCSC genome browser [36]. The curated benchmark was generated using 17,733 human ESTs (including RefSeqs and alternative transcripts) related to 111 genes spread over almost all human chromosomes. Only ESTs mapping onto the corresponding genomic *locus *with a minimum percentage of alignment identity and coverage higher then 80 have been included in the benchmark. This benchmark also includes cases of overlapping and nested genes and can be downloaded at [37]. For each gene we provide the Hugo gene name, the chromosome, the strand, the number of related ESTs and corresponding Genbank accessions.

EasyCluster has been also applied to the complete genome of *Ricinus communis*. In this case, the 4× genome assembly has been downloaded from the JCVI Institute at the following project web page [38]. Ricinus ESTs were retrieved from Genbank using the query (txid3988 [orgn] AND gbdiv_est [prop]).

### Comparative evaluation of EasyCluster

In the comparison with ASmodeler carried out with B1 and B2, EasyCluster was used setting the minimum percentage of alignment identity to 95 and the minimum percentage of alignment coverage to 90. The same settings were used in ASmodeler through the corresponding web page [39].

In the case of benchmark dataset B3, previous EasyCluster settings were both set to 80. The wcd program was downloaded from author's web page and the ClustDB executable for Mac OS X was kindly provided by J. Kleffe. TGICL was retrieved from the Computational Biology and Functional Genomic Laboratory [40] whereas BLASTClust was downloaded from NCBI web site. All tested programs were used with default parameters except for BLASTClust in which the option for the type of input sequences has been switched to nucleic acids.

Evaluation on benchmark datasets has been conducted calculating sensitivity and Jaccard index for each program outcome. Sensitivity is defined as Tp/(Tp+Fn) (Tp = true positives, Fn = false negatives) and gives us an indication of the proportion of true ESTs that has been correctly placed in the correct reference clusters. The Jaccard index instead is defined as Tp/(Tp+Fn+Fp) (Fp = false positives) and measures the similarity between predicted and reference clusters. Type I and Type II error rates have been calculated according to Wang et al. [41].

For the complete genome of *Ricinus communis*, EasyCluster was used setting the minimum percentage of alignment identity to 70 and the minimum percentage of alignment coverage to 50. These less stringent criteria were used because of the lower quality of this dataset exclusively made by EST sequences. Moreover, the alternative splicing option was in effect.

### Implementation

EasyCluster is implemented in the python programming language and can be used on any platform running the python interpreter (at least version 2.4) and GMAP software. For very large EST datasets EasyCluster requires the external *DNA_Stat *library through the *ctypes *module and, thus, version 2.5 of python is strongly recommended. After the installation, EasyCluster can be used by command line or interactively. In the last case, parameters and input file names are entered in response to step by step prompts. Moreover, EasyCluster does not require complex parameters to be set. The user must only provide two multi-fasta files containing genomic and EST sequences and eventually set the minimum percentage of alignment identity and coverage. HTML results can be directly inspected using any web browser although the implemented CSS code has been optimized for FireFox 1.5.X or superior.

## Results

### General features of EasyCluster

Limitations in available software for genome-based EST clustering prompted us to develop a simple new algorithm, implemented in the EasyCluster program. The global procedure is similar to ASmodeler although a number of novelties have been introduced in order to improve the clustering process, provide efficient stand-alone software to analyse own data and make results available for gene prediction and alternative splicing analyses (algorithmic details are provided in Methods section).

A direct comparison between these two systems, therefore, should reveal the effect of novelties introduced in our strategy. Unfortunately, such comparison is not feasible because ASmodeler is implemented in a web service and unable to handle a large number of ESTs. We therefore analysed the same cluster (Hs.122986) considered by Kim et al. [31] in the evaluation of ASmodeler performances. This UniGene entry contains 105 sequences comprising 12 mRNAs and 93 ESTs. We fixed the minimum percentage of alignment identity and coverage to 80% for both programs. ASmodeler detected four clusters including a singleton. The largest group contains 38 sequences mapping in the expected region on chromosome 21. The remaining clusters contain a total of 8 unspliced ESTs and map on the opposing strand in the same genomic region. Only 47 of 105 sequences passed the filtering and were included in the clustering process. EasyCluster generated a single large cluster of 92 sequences in the expected genomic region. Seven unspliced ESTs were discarded because the correct orientation could not be reliably inferred.

We note that EasyCluster not only reconstructed the expected single cluster, but also used a much larger proportion of the input sequences (87%) while using the same filtering criteria. This result, although limited, suggests that our strategy of using GMAP instead of a combination of Blat and sim4 and the subsequent cluster refinement notably improves the cluster quality and incorporates a larger fraction of the available data.

### EST Clustering of human *HOXA *family

EST clustering is particularly challenging for overlapping, nested and paralogous genes. Genome based EST clustering strategies perform better than similarity based EST clustering tools in such cases. We used EasyCluster to build gene-oriented clusters for the human *HOXA *gene family (11 related homeobox genes located on the minus strand of the human chromosome 7). EST, mRNA and RefSeq sequences related to the *HOXA *genomic region were downloaded from UCSC genome browser.

After less then 7 seconds on a desktop computer EasyCluster returned 20 EST clusters including 2 singletons. As expected 11 out of 20 clusters corresponded to *HOXA *genes. We then carried out a careful analysis of extra clusters. In particular, excluding 2 singletons, 5 clusters were located on the plus strand and related to UCSC and putative Gencode protein-coding genes. Another cluster, also located on the plus strand, corresponds to *HOXA11AS, a *non protein-coding gene. Moreover, EasyCluster generated an additional cluster of 13 ESTs on the minus strand upstream of the *HOXA13 *gene. ESTs of this cluster were generated by 5' RACE and might be associated with the promoter region of the *HOXA13 *gene. Finally, singletons though not reliable include 5' ESTs potentially expressed from alternative promoters.

ESTs from *HOXA *family were also clustered by ASmodeler which obtained 22 groups. Major differences between the methods included the presence of 2 additional singletons with ASmodeler, which also places the *HOXA2 *cluster on the wrong strand and the cluster containing 5' RACE ESTs on the opposite strand to EasyCluster.

### Assessment of EasyCluster performance

EasyCluster has been tested on a variety of datasets from different organisms including *Homo sapiens*, *Mus musculus*, *Arabidopsis thaliana *and *Vitis vinifera *(data not shown). However the reliability and quality of the program can only be evaluated using well-established benchmark datasets in which real EST and cDNA sequences are reliably known to be part of a same grouping. At the moment only a limited number of benchmark datasets are available for evaluation purposes even though none are effectively unbiased. For example, the widespread dataset Benchmark10000 generated for the STACK_PACK system at SAMBI Institute and containing the first 10,000 ESTs from the human eye tissue subset has been successfully used to assess the clustering performance of different systems based only on similarity. In this dataset most ESTs are unspliced when mapped onto the current human genome release and thus of limited interest for evaluating genome-based EST clustering systems.

Hazelhurst et al. [24] recently introduced two new benchmark datasets to evaluate the performance and assess the quality of the *wcd *program, an improved version of the *d2_cluster *software [25]. The first dataset called A076941 consists of 76,941 ESTs from *A. thaliana *where reference clusters were generated by assigning ESTs to tentative consensus (TC) sequences of the Arabidopsis Gene Index. The second dataset, is a curated set of 2,294 EST sequences belonging to 34 non-overlapping genes randomly selected from mouse chromosome 4. Unfortunately, neither dataset is suitable for unbiased testing of genome-based EST clustering tools. In the case of the Arabidopsis dataset the reference clusters were generated using TC sequences and could lead to false "real" clusters. On the other hand, the mouse dataset contains many singletons and reference clusters could be more than the 34 indicated due to the BLAST strategy used to generate the set. An obvious drawback of this benchmark is that a similarity-based approach has been used for its construction aimed at the evaluation of a method adopting a similarity-based strategy.

We therefore created a reliable and manually cured benchmark dataset consisting of 17,733 human ESTs and FL-cDNAs from 111 different genes spread on almost all chromosomes. The smallest cluster contains 5 ESTs and the largest 1,090 ESTs (mean 160). Our reference set has been established including for each gene only spliced EST and cDNA sequences showing a minimum percentage of alignment identity and coverage of 80 with the related genomic region. Moreover, the set includes overlapping and nested genes. Our benchmark, therefore, possesses desirable features such as a reliable gene to EST relationship, the inclusion of interesting cases that normally are problematic to cluster and suitability for use to evaluate both genome based and similarity based EST clustering tools.

The quality and global performance of EasyCluster has been so evaluated on our human benchmark dataset by calculating sensitivity and Jaccard index (JI).

The size of our dataset, however, preculdes the use of ASmodeler and BIPASS. Hence, a comparative evaluation with EasyCluster has been restricted to EST clustering tools based on similarity and including wcd, ClustDB, BLASTClust and TGICL. The results of the comparison are shown in Table 1 and indicate that EasyCluster is the most sensitive program even though wcd and TGICL obtained similar levels of sensitivity values. However, in term of similarity to the benchmark, as calculated by JI index, EasyCluster outperforms all other programs.

**Table 1:** Evaluation of different EST clustering tools on our benchmark dataset.

*Program*	*SN*	*JI*	*# Clusters*	*# ESTs per cluster*	α	β
EasyCluster	0.995	0.995	112 (0)	158.3	0.009	0
wcd	0.926	0.797	112 (15)	158.3	0.018	0.045
TGICL	0.906	0.875	125 (0)	141.3	0.144	0.036
ClustDB	0.562	0.424	201 (0)	87.8	0.765	0.009
BLASTClust	0.037	0.037	8255 (7304)	2.1	0.792	0

Sensitivity (Sn), Jaccard Index (JI), number of generated clusters and average number of ESTs per cluster based on our human benchmark dataset. The number of singletons is shown in brackets. Moreover, α and β represents Type I and Type II error rates, respectively.

BLASTClust generated the worst results, with a high number of singletons (70% of all clusters) generated. Results were not substantially improved when sensitivity and JI index were calculated excluding singletons (Sn = 0.085; JI = 0.085).

Taking into account the number of clusters generated and the mean number of ESTs per cluster, wcd and EasyCluster returned the same values even though wcd included 15 singletons.

In Table 1 we report also the Type I and Type II error rates per each program calculated according to Wang et al. [41]. Type I error is a mis-separation error where ESTs from the same gene are erroneously split in two or more clusters (including sigletons). Type II error is, instead, a mis-joining error where two or more non-related ESTs are clustered together. Both error types are very low or absent for EasyCluster. It is clear that for all programs the Type II error rate is not particularly significant. On the contrary, the Type I is instead the most relevant error. This behaviour is probably due to the nature of the benchmark set which does not include paralogous genes – the principal cause of Type II errors. The low Type I error rate in EasyCluster is due to a mis-separation error occurring at the human *HMGB4 *gene currently annotated on the forward strand of the chromosome 1. This gene is nested in an intron of the *CSMD2 *gene annotated on the opposite strand and included in our benchmark. EasyCluster correctly distinguishes ESTs belonging to *CSMD2 *but generates two clusters for *HMGB4*. A cluster contains 20 ESTs and the RefSeq NM_001008728 whereas the second cluster includes 5 ESTs and the RefSeq NM_145205. Both RefSeqs corresponds to *HMGB4 *gene and harbour one intron in the 5' UTR region but no splice site is in common. This clearly leads to two separated clusters. For the same gene, also UniGene provides two clusters Hs.568628 and Hs.667683 even if using additional evidence with respect to EasyCluster (e.g. similarity at protein level).

### EST clustering in *Ricinus communis*

EST clustering is a basic pre-requirement for newly sequenced genomes as groups of related ESTs are typically used in the annotation process. In particular, EST clusters are valuable for gene prediction and detection of alternative splicing events. The contribution to gene finding is manifold as EST clusters can be used to create gene models for the training of *ab initio *systems or as principal evidence sources in different *ab initio *or combined gene prediction tools. Unfortunately, EST clustering for newly sequenced genomes is yet not feasible using available genome-based strategies. The alternative is to switch to similarity-based methods with obvious restrictions, including the parameter setting to optimize the clustering. Moreover, for newly sequenced genomes UniGene entries are often not immediately available. EasyCluster overcomes these limitations providing valuable support to the annotation of any new genome for which a significant number of ESTs has been produced. Recently, the JCVI Institute (J. Craig Venter Institute) released a 4× genome draft of the plant *Ricinus communis*. This plant is significant for the production of oil and there is thus a direct interest in the characterization of its genetic resources. The genome draft of *Ricinus *(estimated to be 320 Mb) was downloaded from the JCVI Institute and more than 57,000 ESTs were retrieved from GenBank. EasyCluster was used to build the first compilation of EST gene-oriented clusters for *Ricinus communis*. Main results summarizing our analysis are reported in Table 2. EasyCluster generated 5,879 clusters in 32 minutes (including GMAP run and database building) using 57,690 ESTs mapping onto 803 different contigs. As expected, 50% of all generated clusters were singletons and more than 32% contained at least 3 ESTs. In diverse cases, clusters were partially overlapping and could represent unique transcribed *loci*. The lack of additional evidence, however, prevents more accurate cluster refinement.

**Table 2:** EasyCluster statistics and results for *Ricinus communis*

#ESTs	#Ex_ESTs	#Unique ESTs	#Unspliced ESTs	#Used ESTs	#Clusters	#Singletons
57690	482	33907	19921	35272	5879	2944

For EST clustering in *Ricinus communis*, EasyCluster used 57,690 ESTs. A reduced number of ESTs (Ex_ESTs) have been excluded due to poor genomic mapping and 19,921 ESTs have been filtered out since unspliced and with no defined orientation. Finally, 5,879 clusters have been generated using 35,272 high quality ESTs.

**Figure 2:**
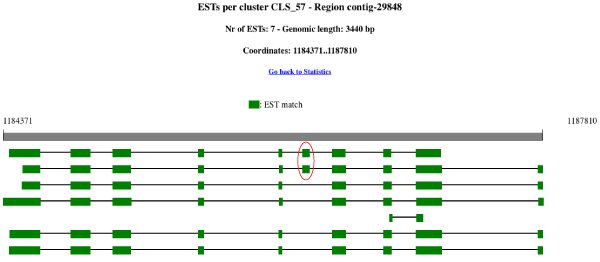
**Example of EasyCluster graphical report per cluster**. This figure shows a graphical overview of an EST cluster generated by EasyCluster on *Ricinus communis *genome. The cluster comprises 7 ESTs mapping on the *Ricinus *contig number 29848 and 3440 bp long. For each EST the exon/intron structure is shown in green squares joined to black lines. The red circle underlines an exon skipping event occurring in the first two ESTs.

Despite the relatively low number of available ESTs, EasyCluster was also able to predict 918 alternative splicing events. An exon skipping example is shown in Figure 2 and the number of events per alternative splicing category is in Table 3. According to EasyCluster results, the intron retention event is the most frequent accounting for 30% of all detected events. The alternative acceptor is the second most common event. Our results are in line with current findings about the impact of alternative splicing in plants, in which intron retention is generally considered the most frequent event [42].

**Table 3:** Alternative splicing events in *Ricinus communis*.

All events	Alt_acc	Alt_don	Skip	IR	Others
918	254	187	92	277	108

Distribution of the 918 alternative splicing events deduced in *Ricinus communis*. Alt_acc: alternative acceptor; Alt_don: alternative donor; Skip: exon skipping; IR: intron retention; Others: combination of simple events (ex.: cassette exons).

## Discussion

ESTs represent an invaluable resource for gene discovery, gene mapping, genome annotation, SNP discovery and alternative splicing detection. Over the last few years, great efforts have been made in clustering EST data in order to group together sequences deriving from the same gene. Currently, similarity-based methods represent the main approach to address this task, especially when no additional genomic evidence is available. Such methods, however, suffer from notable limitations. Indeed, ESTs from paralogous genes or from nested and overlapping genes may not be correctly clustered. These problematic cases can be partially or completely resolved unsing genome-based EST clustering tools. Unfortunately, a very limited number of these programs have been yet developed. UniGene, for example, is only available as database and no software has been released publicly until now [21]. Probably the complex pipeline behind the UniGene methodology precludes the implementation of a stand-alone program. On the other hand, ASmodeler [31] and BIPASS [28] are two web services devised with the aim to provide tools to analyse the alternative splicing pattern. Therefore, they implement EST clustering procedures even though the clustering process is not the goal but an intermediate pipeline step to study alternative splicing. The lack of dedicated software to generate gene-oriented EST clusters using genomic information stimulated to develop a specialized tool in this direction. Reliable EST clusters can be used for a variety of purposes including alternative splicing analysis and gene annotation. This last task is mainly true for newly sequenced genomes where limited training sets cannot provide a solid support to *ab initio *gene prediction. Clusters of genomically aligned ESTs, instead, can be used as strong evidence sources significantly improving gene-finding procedures. Given the nature of ESTs, tissue specific sequences can also be clustered to discover peculiar transcript variants providing fruitful insights in gene expression.

The EasyCluster program described here has been devised to take advantage of genomic mapping. ESTs and FL-cDNAs are first aligned to a reference genome by the state-of-the-art tool GMAP supplying mapping information and splice site detection at the same time [32]. Genomically aligned ESTs are subsequently clustered according to the reasonable biological assumption that two or more ESTs are part of the same transcribed *locus *if they share at least one splice site. We maintain that in absence of additional evidence this criterion is the most biologically plausible and meaningful: it is also in line with recently proposed operational definitions of genes [43,44]. Obvious limitations can be inferred considering the error prone nature of ESTs and their limited lengths. Such sequences can partially cover transcribed *loci *leading to multiple clusters per gene. In the plant *Ricinus communis*, for instance, we obtained EST clusters that are overlapping at their ends but without common splice sites. These clusters could represent single genes and should be merged. However, the overlap could involve untranslated regions of different neighbour genes and thus the merging could introduce clustering errors. In order to reduce this kind of errors EasyCluster provides unmerged clusters in absence potentially relevant biological evidence. A limiting issue of the methodology, through not so obvious, concerns the managing of ESTs mapping with multiple and/or identical paths onto a reference genome. These multiple hits due to paralogous genes from recent duplication events are difficult to handle and in all cases in which discerning the most significant alignment is not feasible, all detected paths are included in the clustering procedure. As a consequence, alternative paths tend to increase singletons that could be recognized as in a meaningless post-processing phase. Clusters containing only one EST, can be due to mapping errors (especially if the EST quality is low) or to a low number of available expressed sequences. Both situations could explain the high number of singletons obtained in *Ricinus *for which only 57,690 EST sequences of different quality are stored in the public dbEST database [4].

EasyCluster comes with valuable benefits for users giving the opportunity to evaluate the effect of adding or removing specific ESTs and graphically explore generated clusters supplying extra facilities not implemented in other tools as UniGene. EasyCluster is not web-limited and the whole clustering procedure can be completed on desktop computers or on any computer in which GMAP can be installed. Moreover, the interactive modality facilitates the use of the program to people not completely familiar with command-line based software.

As shown in Results section, EasyCluster can be used for distinct purposes. Interested users, for example, use the program to refine and/or visualize UniGene clusters, generate EST clusters in organisms where UniGene information are not available or to build EST groups for paralogous genes. In the *HOXA locus *example, we demonstrate that our clustering procedure can also provide reliable genomic annotations predicting the presence of transcribed units compatible with available ENCODE findings [45].

Previous works on EST clustering addressed the reliability and performance of proposed methodologies using very large EST datasets or comparing the clustering with the corresponding obtained by other systems. Large datasets are useful to assess the performance of a method in terms of speed and code stability. However, the comparison strategies used are questionable. For these large datasets, the true coposition of clusters is unknown and thus the resulting comparisons and evaluations lead to mere speculations. To assess the quality of EasyCluster, we introduced a new benchmark dataset consisting of 17,733 human ESTs derived from 111 different genes including overlapping and nested examples. This benchmark represents a valid test to demonstrate the reliability of the method and the basic requirement that ESTs directly derived from a given gene have to be addressed only towards that specific gene. Moreover, by using available clustering software we show that genomic information can lead better results than similarity-based methods, reducing Type I (mis-separation) and Type II (mis-joining) errors. At the moment only the wcd program [24] is able to provide results comparable with ours. However, it should be considered that BLASTClust [18] and ClustDB [27] are not specifically designed to cluster ESTs, though they can handle this kind of data and run in a very efficient way. ClustDB, for instance, was able to cluster our benchmark data in only 20 seconds. EasyCluster, instead, completed the clustering (including the mapping step) in less then 6 min like TGICL [19], whereas wcd took 7 min and 6 sec. All programs were run on a Linux server using only one CPU. It should also be considered that run parameters can have a significant impact on results and in our tests all programs were used with default options and thus running sub-optimally. However, the choice of optimal parameters is not a trivial task and in similarity-based EST clustering tools they should be optimized according to species-specific test sets. In EasyCluster, instead, only two simple and intuitive parameters must be set to filter out unwanted ESTs according to the percentage of alignment identity and coverage. For each generated cluster, EasyCluster can also provide the prediction of alternative splicing events taking into account genomic coordinates of mapped ESTs and thanks to the implementation of an ASTALAVISTA-like algorithm [34].

The flexibility of EasyCluster, coupled with the possibility of handling large numbers of ESTs, makes our software ready for genome-wide applications and a valid alternative to the clustering of transcriptome data from the next generation of pyrosequencing reads. Moreover, the organization and structure of our python code in modules makes simple the implementation of new or existing algorithms to improve cluster refinement or assemble full-length transcripts per cluster. A final advantage of EasyCluster is the output format consistent with the standard GFF format [35], easy to parse and use in a variety of computational programs such as the *Cluster_merge *script by Eyras et al. [46]. Lists of ESTs per cluster can also be extracted in Fasta format using the additional *getFastas.py *script (currently part of EasyCluster release) enabling the use of assembling software such as CAP3 [16].

## Conclusion

To our knowledge, EasyCluster is the first free available software to generate gene-oriented clusters of ESTs using genomic information. It uses GMAP to efficiently map ESTs onto a reference genomic sequence. The clustering procedure is based on the biological assumption that ESTs related to a specific spliced gene share at least one splice site. EasyCluster avoids and overcomes existing clustering limitations due to nested and overlapping genes. Depending on mapping, it can also reliably distinguish paralogous genes. EasyCluster also implements the detection of alternative splicing. Since it represents a hot topic in genomics as well as transcriptomics analysis, EasyCluster will be improved in order to assemble full-length transcripts per cluster and provide provisional functional annotation. The use of additional external evidence will also be permitted in the cluster refinement phase. Moreover, graphical overviews will be created by SVG in order to make the HTML code more stable for all available web browsers.

## Availability and requirements

Project name: EasyCluster

Project home page: 

Operating system: Platform independent

Programming language: Python

Other requirements: GMAP

License: GNU GPL

Restrictions: none

## Competing interests

The authors declare that they have no competing interests.

## Authors' contributions

EP developed the software, performed the clustering evaluation and drafted the manuscript. FM provided EST datasets including the manually cured human benchmark. GP formulated the objectives of the project and supervised the development. All authors read and approved the final manuscript.
